# Patterns of prescription of antipsychotics in Qatar

**DOI:** 10.1371/journal.pone.0241986

**Published:** 2020-11-09

**Authors:** Sami Ouanes, Imen Becetti, Suhaila Ghuloum, Samer Hammoudeh, Mena Shehata, Hany Ghabrash, Areej Yehya, Hawra Al-Lawati, Nora Al-Fakhri, Huma Iram, Nighat Ajmal, Yassin Eltorki, Hassen Al-Amin

**Affiliations:** 1 Department of Psychiatry, Hamad Medical Corporation, Doha, Qatar; 2 Weill Cornell Medical College, Medical Education, Doha, Qatar; 3 Research Department, Weill Cornell Medical College, Doha, Qatar; 4 Pharmacy Department, Hamad Medical Corporation, Psychiatry Hospital, Doha, Qatar; 5 Psychiatry Department, Weill Cornell Medical College, Doha, Qatar; University of Toronto, CANADA

## Abstract

**Objective:**

Even though all guidelines recommend generally against antipsychotic polypharmacy, antipsychotic polypharmacy appears to be a very common practice across the globe. This study aimed to examine the prescription patterns of antipsychotics in Qatar, in comparison with the international guidelines, and to scrutinize the sociodemographic and clinical features associated with antipsychotic polypharmacy.

**Methods:**

All the medical records of all the inpatients and outpatients treated by antipsychotics at the Department of Psychiatry–Hamad Medical Corporation (HMC) in Doha, Qatar (between October 2012 and April 2014) were retrospectively analyzed. We retrieved the available sociodemographic data, psychiatric features, and details on the medication history.

**Results:**

Our sample consisted of 537 individuals on antipsychotics (2/3 were male; mean age 33.8±10.2 years), prescribed for a psychotic disorder in 57%, a mood disorder in 9.3%, and various other diagnoses in 33.7%. About 55.9% received one antipsychotic, 29.6% received two antipsychotics, and 14.5% received more than two antipsychotics. Polypharmacy was associated with younger age (p = 0.025), being single (p<0.001), the diagnosis of a psychotic disorder (p<0.001), and previous admissions to psychiatry (p<0.001).

**Conclusions:**

Antipsychotic polypharmacy appears to be quite common in Qatar, as it is the case in many other countries, in contrast with most international recommendations. Studies are needed to explore the reasons behind this disparity.

## Introduction

The international prescribing guidelines for the treatment of schizophrenia recommend the use of antipsychotic (AP) monotherapy, both for the acute and maintenance phases of schizophrenia [1, 2]. AP polypharmacy has been associated with a tremendous increase in the treatment cost [3], more side effects, including sedation, extrapyramidal symptoms, metabolic changes, cardiovascular complications [4], and even with increased mortality [5]. Simultaneously, there is no robust evidence to support that AP polypharmacy may bring about substantial clinical benefits in terms of residual psychotic symptoms [6]. However, one cohort study showed that AP polypharmacy was associated with a 10% lower relative risk of rehospitalization. Yet, the AP combinations that were associated with the lowest risk of rehospitalization mostly included clozapine, and clozapine monotherapy was superior to most AP combinations, not including clozapine [7].

AP polypharmacy appears to be a widespread practice across the globe [8, 9]. According to a meta-analysis by Gallego *et al*., the median prevalence of AP polypharmacy was 19.6% (interquartile range, IQR = 22.1%) [10]. This trend is even appearing to accentuate in many regions of the world [11], and the few studies from the Middle East region have shown high rates of AP polypharmacy in Egypt, Saudi Arabia, the United Arab Emirates (UAE), and Qatar [12, 13]. In contrast with the international recommendations, these high AP polypharmacy rates indicate that the available pharmacological interventions and treatment guidelines may still be far from meeting all the needs in the clinical practice [14]. Some clinicians justify the use of AP polypharmacy to improve the AP efficacy, by enhancing the dopamine 2 (D2) receptor blockade, or by targeting several receptor sites in addition to D2 receptors. In contrast, others believe that AP polypharmacy may achieve more rapid control of acute symptoms [3, 15, 16]. Generally, these practices have more to do with empirical judgment rather than evidence-based reasoning [3]. Hence, choosing AP polypharmacy rather than monotherapy appears to be too prone to cognitive biases, including notably self-serving bias [17].

Proper high-quality studies about AP polypharmacy are mostly lacking, and research on the topic remains sporadic. There is an apparent lack of commercial incentive for any drug company to examine its manufactured drug's effects when added to a competitor’s drug. Moreover, there is little academic interest in studying complex AP polypharmacy cases since the interpretation of the results might be daunting [3]. Studies from the Middle-Eastern region are even more scarce [12, 13]. Evaluating the local patterns of AP prescription and its associated sociodemographic and clinical factors may help improve drug safety and effectiveness [9]. A previous study in Qatar showed a high prevalence of AP polypharmacy, but the study only included inpatients for six months and was purely descriptive [13]. Thus, the aims of this more extensive study were (1) to examine the prescription patterns of APs in Qatar, in comparison with the international guidelines, including inpatients and outpatients; and (2) to explore the sociodemographic and clinical features associated with AP polypharmacy (as the previous study did not analyze those).

## Methods

### Participants and setting

This retrospective study is part of a project assessing the biopsychosocial aspects of patients receiving antipsychotics in Qatar. This paper focuses on the patterns of prescription of APs at the Department of Psychiatry in Hamad Medical Corporation (HMC). The Department of Psychiatry and the Mental Health Hospital at HMC are the major providers of inpatient and outpatient psychiatric care in Qatar, including the dispensing of psychotropics. The hospital includes four inpatient wards (total of 70 beds) with an average of 95% occupancy and ten outpatient clinics that receive about 120 visits per working day. The most common diagnoses at the outpatient clinics include mood disorders, anxiety, stress-related disorders, substance misuse, and schizophrenia. The study was carried out between June 2014 and June 2016, retrospectively collecting data about antipsychotic use between October 2012 and April 2014.

### Ethical considerations

The institutional review committees at both the Medical Research Center of HMC and Weill Cornell Medicine in Qatar (WCM-Q) approved this project. The study was exempted from the written informed consent as we did not contact the subjects, and the data collected from the records was de-identified.

### Sampling method and sample size determination

We followed convenience sampling and retrieved the records of all patients (age above 18 years) who received APs (for more than one week) at the Department of Psychiatry in HMC during the period between October 2012 and April 2014. No sample size calculation was required, given that we included all cases during the chosen period. A total of 645 records were identified, but only 537 cases were included in the study after excluding patients who received APs for only one week or less.

### Study design and measures

A set of questionnaires were designed to collect the following information: socio-demographics, clinical features related to the psychiatric history (as documented by the treating psychiatrist in the patients’ medical records), as well as the details concerning the psychotropic medications. The prescribed psychotropic medications were retrieved from the records, including the type, the dose, and the duration of each prescribed psychotropic drug. For antipsychotics, chlorpromazine-equivalent doses were calculated [18]. We examined the medications prescribed for the last six months before the date of assessment. These questionnaires were piloted and validated using a sample of 20 records before collecting the research data. All raters used the same manual that was developed to code the data retrieved from the files. Ten assessors (one postdoctoral researcher in public health, one medical doctor, one nurse, one clinical psychologist, three psychiatry residents, and three medical students) were involved in collecting the different sections of the questionnaires for this project. All raters received proper training and supervision before obtaining the actual data. Two raters independently assessed 100 records to ensure excellent inter-rater reliability. The psychiatric diagnosis was collected from the records according to the treating psychiatrists who mostly used the Diagnostic and Statistical Manual of Mental Disorders (DSM-TR-IV) criteria for diagnosis [19].

### Statistical analysis

The statistical analysis was conducted using the Statistical Package for the Social Sciences (IBM-SPSS, version 23.0, IBM Corp). A p-value of 0.05 or less was considered statistically significant. For descriptive statistics, we calculated the frequency for categorical variables, and the mean and standard deviation (SD) for the continuous ones. The patients were divided into two groups: AP monotherapy and AP polypharmacy (use of two or more APs, each for more than one week). The differences between these groups were analyzed using a chi-square test for categorical variables and t-test for the continuous ones. We constructed a multiple logistic regression model, with the AP monotherapy vs. polypharmacy as the dependent variable, and with age, gender, marital status, history of previous admission(s) to psychiatry, diagnosis, duration of illness, as well as the clinical sets of symptoms (delusions, hallucinations, thought disorder, catatonia, negative symptoms) as independent variables.

## Results

### Sociodemographic and clinical characteristics of the study sample (Table 1)

Our sample consisted of 537 individuals on AP medication. The participants were from different nationalities (including 25.3% Qataris, 20.1% non-Qatari Arabs, 33% from South Asia). About 2/3 were male, and the mean age was 33.8±10.2 years (Table 1). The AP medication was prescribed for a psychotic disorder in 57%, and a mood disorder in 9.3%. Around one third had various other diagnoses, including anxiety disorders, personality disorders, and substance use disorders. The most common symptoms observed in patients on APs were hallucinations (52.0%), delusions (47.4%), and thought disorder (29.6%). The mean duration of illness (in years) was 4.6±6.4 (Table 1).

**Table 1:** Sociodemographic and clinical characteristics.

**Gender, n(%)**	Male	349(65.0)
	Female	188(35.0)
**Age, years (m ±SD)**^**a**^	33.8±10.2	
**Nationality, n(%)**	Qatar	136(25.3)
	Arab (non-Qatari)	108(20.1)
	South Asia	177(33.0)
	Filipino	41(7.6)
	Other	75(14.0)
**Marital status, n(%)**	Married	259(48.8)
	Single	237(44.6)
	Divorced/Widowed	35(6.6)
**Diagnosis, n(%)**	Brief psychotic /Schizophreniform disorder	132(24.6)
	Schizophrenia	161(30.0)
	Other psychotic disorders	13(2.4)
	Bipolar disorder	40(7.4)
	Major depressive disorder	10(1.9)
	Anxiety disorder	8(1.5%)
	Substance use disorder	18(3.4)
	Other^b^	155(28.8)
**Duration of illness, years (m ±SD)**	4.6±6.4	
**Number of hospitalizations, n(%)**	None	296(55.1)
	1	106(19.7)
	2 to 4	59(11.0)
	5 or more	76(14.2)
**Symptoms, n(%)**	Delusions	255(47.4)
	Hallucinations	279(52.0)
	Thought disorder	159(29.6)
	Catatonic symptoms	40(7.5)
	Negative symptoms	174(32.4)

^a^ m: Mean; SD: Standard Deviation.

^b^ Other diagnoses include obsessive compulsive disorder, eating disorders, personality disorders, and non- specified diagnoses.

### Antipsychotic prescription patterns (Table 2)

Around 44.1% (n = 237) of the patients on APs were on more than one AP: 29.6% (n = 159) were on two APs, 10.4% (n = 56) on three APs, and 4.2% (n = 22) were on four APs or more. Among patients with a psychotic disorder, the prevalence of AP polypharmacy was 58.8% (n = 200), with 15% (n = 46) on three APs or more. The mean number of antipsychotics prescribed was 1.6±0.9. The mean AP chlorpromazine-equivalent dose was 577.8±736.4, with values ranging between 29 and 4878mg. In patients with a psychotic disorder, the mean chlorpromazine-equivalent dose was 577.8±736.4mg. More than half (56.2%) were on second-generation APs (SGA), 15.5% on first-generation APs (FGA), whereas 28.3% were on a combination of FGAs and SGAs. Out of the patients on clozapine (n = 13; 2.4%), all but one were on at least another antipsychotic. The psychotropic drugs most commonly prescribed with APs were antidepressants (34.6%; n = 186), followed by mood stabilizers (13.2%; n = 71), and benzodiazepines (12.5%; n = 67). Selective serotonin reuptake inhibitors accounted for 87.1% of the antidepressants prescribed (n = 162). Among mood stabilizers, anticonvulsants were by far more commonly prescribed (98.5%; n = 70) than lithium (1.5%; n = 1) (Table 2). The median time before switching or adding a new AP was around ten days.

**Table 2:** Description of antipsychotic prescription patterns.

**Number of prescribed antipsychotics, %**	One antipsychotic	300(55.9)
	Two antipsychotics	159(29.6)
	More than two antipsychotics	78(14.5)
**Antipsychotic dose, chlorpromazine equivalent (m ±SD)**^**a**^	577.8±736.4	
**Antipsychotic generation, % patients**	First-generation	235(43.8)
	Second-generation	454(84.5)
**Antipsychotic medication, % patients**	Amisulpride	2(0.4)
	Aripiprazole	32(6.0)
	Chlorpromazine	11.0%
	Clozapine	13(2.4)
	Flupenthixol	34(6.4)
	Fluphenazine	7(1.3)
	Haloperidol	109(20.3)
	Olanzapine	246(45.9)
	Paliperidone	20(3.7)
	Quetiapine	109(20.3)
	Risperidone	156(29.1)
	Trifluoperazine	63(11.7)
	Zuclopenthixol	9(1.7)
**Associated psychotropic medication, % patients**	Antidepressants	186(34.6)
	Benzodiazepines	67(12.5)
	Mood stabilizers	71(13.2)
	Antihistamines	26(4.8)
	Anticholinergics	77(14.4)

^a^ m: Mean; SD: Standard Deviation

### Associations between sociodemographic and clinical variables and the number of AP prescribed

Table 3 shows the statistical associations between the sociodemographic and clinical variables with the number of AP prescribed. In univariate analysis (as the first step in our comparative analysis between AP monotherapy and AP polypharmacy), sociodemographic factors that were associated with AP polypharmacy were younger age (p = 0.025) and being non-married (p<0.001). In contrast, the nationality and the gender did not show any association with the AP prescription pattern. Clinical factors linked to AP polypharmacy included: a diagnosis of a psychotic disorder (p<0.001), previous admission to psychiatry (p<0.001), as well as the presence of delusions (p<0.001), hallucinations (<0.001), and thought disorder (p = 0.002).

**Table 3:** Associations between sociodemographic and clinical variables and the number of AP prescribed.

Variable	Antipsychotic monotherapy group (n = 300)	Antipsychotic polypharmacy group (n = 237)	p
**Gender, n(%) male**	197(65.7)	152(64.1)	0.712^+^
**Age, years** (m ±SD)^a^	34.7±10.9	32.7±9.2	0.025^x^
**Origin, n(%) Qatari**	71(23.7)	65(27.4)	0.320^+^
**Origin, n(%) Arab**	129(43.0)	115(48.5)	0.202^+^
**Marital status, % Married**	170(57.0)	89(38.2)	<0.001^+^
**Diagnosis, % psychotic disorder**	126(42.0)	180(75.9)	<0.001^+^
**Duration of illness, years** (m ±SD)	4.6±7.2	4.5±5.5	0.898^x^
**Previous admission(s) to psychiatry, n(%)**	111(37.0)	130(54.9)	<0.001^+^
**Delusions, n(%)**	114(38.0)	141(59.5)	<0.001^+^
**Hallucinations, n(%)**	130(43.4)	149(62.9)	<0.001^+^
**Thought disorder, n(%)**	70(23.3)	89(37.6)	0.002^+^
**Catatonic symptoms, n(%)**	12(4.0)	28(11.8)	0.065^+^
**Negative symptoms, n(%)**	105(35.0)	69(29.1)	0.362^+^

^+^Chi square test

^x^ T- test

^a^ m: mean; SD: standard deviation.

The multiple logistic regression model (as the second step in our comparative analysis between AP monotherapy and AP polypharmacy) (Table 4) had a Nagelkerke R Square of 0.5, which validates the adequacy of the model. The outcome was AP monotherapy vs. AP polypharmacy, and the predictors entered in the model are listed in Table 4. The model showed that AP polypharmacy was independently associated (after controlling for other relevant covariates) with the diagnosis of a psychotic disorder (p = 0.002) with an odds ratio (OR) = 12.221[95%CI: 2.458–60.767], and with the female gender (p = 0.041; OR = 6.525[95%CI: 1.075–39.612]).

**Table 4:** Factors associated with antipsychotic polytherapy—multiple logistic regression.

Variable	OR^a^	OR 95% CI^b^	p
**Age**	0.929	0.842–1.025	0.140
**Gender, female**	6.525	1.075–39.612	0.041
**Marital status, married**	0.199	0.038–1.054	0.058
**Previous admission(s) to psychiatry**	1.745	0.376–8.098	0.477
**Diagnosis, psychotic disorder**	12.221	2.458–60.767	0.002
**Duration of illness**	1.050	0.912–1.210	0.495
**Delusions**	1.131	0.252–5.084	0.872
**Hallucinations**	0.561	0.133–2.372	0.432
**Thought disorder**	1.085	0.263–4.471	0.911
**Catatonia**	0.000	-	0.999
**Negative Symptoms**	1.153	0.153–8.678	0.890

^a^ OR: Odds ratio

^b^ 95% CI: 95% confidence interval

## Discussion

In this study about AP prescription patterns in Qatar, we found a high rate of AP polypharmacy (44.1%), with 14.5% even receiving three APs or more. SGAs were used more often than FGAs (84.5% vs. 43.8%). AP polypharmacy was associated with the female gender, as well as the diagnosis of a psychotic disorder.

### Prevalence of antipsychotic polypharmacy

The prevalence of AP polypharmacy reported in different studies can vary considerably depending on many factors, including the exact definition of AP polypharmacy (the requirement of minimum duration and/or dosage), the sociodemographic characteristics (age, gender), the clinical diagnoses included, the clinical setting (outpatient vs. inpatient), and also significant regional differences [9, 20]. The prevalence we found in our study (44.1%) is actually the same as the prevalence reported in a previous study in Qatar about inpatients in 2012 [13], even though our study also included outpatients. The rate of AP polypharmacy we found was higher than the median of 19.6% reported by the meta-analysis by Gallego *et al*., and even higher than the third quartile of 35.0% [10]. The AP polypharmacy rate in our study can be considered relatively high, especially when we take into consideration that we included all patients on APs regardless of the underlying diagnosis, while patients included in the meta-analysis had mainly schizophrenia (82.9%) [10]. The prevalence of AP polypharmacy in patients with a psychotic disorder (58.8%) was even higher than in the whole sample.

AP polypharmacy rate in Qatar seems to be more in line with the figures in Asia (32%; IQR = 19.2–53.0%), rather than in North America (16%; IQR = 7.2–24.4%), or Europe (23%; IQR = 15.0–42.1%) [10]. Indeed, the fourth survey of Research on Asian Prescription Patterns on APs reported overall AP polypharmacy rates of 42.2±12.0, with rates being as high as 59.1% in Vietnam, 55.0% in Japan, and 54.3% in Thailand [21]. Likewise, Xue *et al*. found that 43.9% of inpatients with schizophrenia in Wuxi and Wuhan, China was on AP polypharmacy [9]. Similarly, in a cross-sectional study in seven hospitals from four regions in Japan, the mean number of APs prescribed to patients with schizophrenia was 1.76±0.86, comparable to our figures (1.6±0.9) [22]. A multicenter, observational study conducted in Egypt, Kuwait, Saudi Arabia, and the UAE and found that SGAs were used in inpatients with schizophrenia in combination with other SGAs or FGAs in 65.5% of the cases [12]. However, the population (acute inpatients with schizophrenia) may partly explain why the reported rates were higher than in our study. In Qatar, the fact that most of the medical costs are covered by the State, rather than by private insurances, could contribute to this high rate of AP polypharmacy, at least among Qataris [9].

### Type and dose of antipsychotics prescribed

We found that the rate of SGA prescription was quite high in Qatar (84.5%), a figure that did not differ much from similar studies performed in other Arab countries (95.6%) [12], China (86.6%) [9], Korea (93%) [8], New Zealand (87.0%) [23], or Turkey (96.9%) [24]. This high rate of SGA prescription follows the international trend. It may be due to the increased availability of newer antipsychotics and the conviction that SGAs produce fewer extrapyramidal and cognitive side effects [8, 12].

However, the rate of prescription of FGAs was relatively high (43.8%), when compared to rates reported in studies performed in the other Gulf States: Saudi Arabia, Kuwait, UAE (23.4%) [12], Korea (7%) [8], New Zealand (13.0%) [23], or Turkey (17.2%) [24]. This high rate for the FGAs is rather intriguing because the public health system in Qatar and the full availability of many SGAs in the country might offset some of the significant benefits of FGAs over SGAs, namely cost and availability in specific settings. One of the possible explanations might be that FGAs are often used to augment SGAs. Still, this strategy can sabotage many of the benefits of using SGAs over FGAs in terms of extrapyramidal and cognitive side effects [3].

Despite the higher rate of AP polypharmacy in our sample, the mean chlorpromazine equivalent dose (577.8mg) was lower than in other studies: 684.1mg in the study by Yazici *et al*. [24], or 732.1mg in the study by Kim *et al*. [8]. This lower dose may suggest a tendency in Qatar towards combining small doses of multiple APs, rather than optimizing/maximizing the dose of one AP.

Besides, the rate of clozapine prescription in our study was also low (2.4%), similar to that in the United States (2.5%) [25], yet clearly inferior to the mean rate of clozapine prescription across the world between 2000 and 2009 (13.6%) [10]. This rate is also lower than that of prescription reported in a Chinese study among inpatients with schizophrenia (44.0%) [9], and even lower than the rate of clozapine prescription reported in a British study among patients with first episode psychosis (8.3%) [26]. It is quite established that clozapine may have an indication in up to 30% of patients with schizophrenia [25]. Clozapine is also the only evidence-based option in treatment-resistant schizophrenia, yet remains particularly underutilized worldwide [27]. This underutilization is seemingly even more pronounced in Qatar.

The presence of contraindications to clozapine in individual patients, as well as the reluctance of some patients to take it (given the mandatory blood monitoring), may explain a portion of the underuse. Still, the resistance of the psychiatrists themselves towards prescribing clozapine likely accounts for most of the underuse, worldwide [28], as well as in the Arabian Gulf region [29]. The low rate of clozapine use in Qatar, contrasting with the high rate of use of two or even more APs, also suggests that AP polypharmacy is likely used as an alternative to clozapine, despite the absence of evidence to support this practice [30].

### Sociodemographic and clinical features associated with AP polypharmacy

We found that only the female gender and the diagnosis of a psychotic disorder (rather than other diagnoses for which APs were prescribed) were independently associated with AP polypharmacy (in multiple regression analysis). Other studies did not report any particular associations with gender or age [8, 10, 15, 24]. The diagnosis of schizophrenia has often been an independent predictor of AP polypharmacy in previous studies [10, 15]. Other clinical variables that were found to be associated with AP polypharmacy include: inpatient setting or history of previous admissions [10, 15], longer duration of illness [8], more severe symptoms [24], in particular, more severe positive symptoms [8], lower social functioning [8], the use of depot APs [8], use of FGAs [10], use of mood stabilizers [15], as well as use of anxiolytics [15].

Given that most studies are cross-sectional or retrospective, it is often difficult to determine the direction of these associations between AP polypharmacy, disease severity, and poor social functioning. Indeed, while it may seem evident that the most severe cases would be more likely to receive more than one AP, AP polypharmacy (especially when including FGAs) may also worsen cognitive and negative symptoms, leading to worse social functioning [31].

### Strengths and limitations

The main strengths of the present study include: (a) this is one of the first studies about AP prescription patterns in the Arab and Middle Eastern regions. (b) as HMC is by far the largest provider of psychiatric care in the country, we can safely assume that most patients on APs in Qatar were included at the time of the study. On the other hand, some limitations need to be mentioned: First, due to the retrospective nature of the study, the direction of the statistical associations found could not be determined. Second, some of the data (notably reasons behind AP switching or augmentation) were missing. Third, the study did not use psychometric scales to quantify positive, negative, and cognitive symptoms. Associations between the different clusters of symptoms and AP prescription patterns (including AP polypharmacy and the use of SGAs vs. FGAs) could have added value to the study. Fourth, the study assessed the antipsychotic prescription patterns a few years ago, and thus our findings might not reflect the current practices.

## Conclusions

AP polypharmacy appears to be quite common in Qatar, as it is the case in many other countries, contrasting with most international recommendations. Very few studies explored the reasons behind this disparity; it is possible that many clinicians prefer using polypharmacy over the resort to higher doses of one single AP or the use of clozapine. Further monitoring studies in Qatar are needed to investigate whether the AP polypharmacy trend is rising or in the fall. The development of medication algorithms can help rationalize the usage of APs in general since many of the prescriptions are off-label, and safer alternatives might be available, and to limit the use of AP polypharmacy to particular clinical scenarios where no better (more evidence-based or safer) choices are possible.
